# Short-, Medium- and Long-Term Metabolic Responses of Adult Meat Ewes Subjected to Nutritional and β-Adrenergic Challenges

**DOI:** 10.3390/ani10081320

**Published:** 2020-07-30

**Authors:** Eliel González-García, Moutaz Alhamada, Nathalie Debus, Jean-Baptiste Menassol, Anne Tesnière, Jéssica Gonçalves Vero, Bruna Barboza, François Bocquier

**Affiliations:** 1 INRAE UMR868, Systèmes d’Elevage Méditerranées et Tropicaux (SELMET), CEDEX 1, F-34000 Montpellier, France; moutaz.alhamada@hotmail.com (M.A.); nathalie.debus@inrae.fr (N.D.); jean-baptiste.menassol@supagro.fr (J.-B.M.); anne.tesniere@inrae.fr (A.T.); francois.bocquier@supagro.fr (F.B.); 2 L’institut Agro-Montpellier SupAgro, Sciences Animales, Department MPRS, CEDEX 1, F-34000 Montpellier, France; 3 Universidade Estadual de Londrina (UEL), Centro de Ciências Agrárias, Londrina CEP 86057-970, Brazil; jgveroo@gmail.com (J.G.V.); barbozabruna1@gmail.com (B.B.)

**Keywords:** metabolic adaptation, adaptive capacity, undernutrition, feed shortage challenge

## Abstract

**Simple Summary:**

The ability of *Merinos d’Arles* ewes to quickly overcome undernutrition situations by efficiently using their body energy reserves was confirmed in this study. There is potential for a simplified ß-adrenergic challenge protocol helping to identify differences in adaptive capacity among individuals reared and fed under similar conditions in the same flock.

**Abstract:**

Shortage and refeeding situations lead to switches in metabolic pathways induced by undernutrition and body energy reserve (BR) replenishment cycles. In a 122-d experiment, 36 adult *Merinos d’Arles* ewes were chosen and first accustomed to diet ingredients (i.e., wheat straw, pelleted alfalfa and sugar beet pulp) and the facility environment for 22 d. Then, ewes were randomly assigned to one of three “diet challenge” treatments during 50 d, (control, underfed and overfed; 12 ewes each) corresponding to 100%, 70% or 160% of energy requirements allowances, respectively. Then, a “refeeding challenge” was applied the last 50 d (i.e., diets adjusted with the same ingredients). An individual monitoring of body weight (BW), body condition score (BCS) and energy metabolism was carried out. The last day, a “ß-adrenergic challenge” was applied. Anabolic or catabolic responses were accompanied by synchronized metabolic regulations, leading to contrasting metabolic and BR profiles. Average BW and BCS were higher and lower in overfed and underfed ewes, respectively, which was proportional to lower and higher BR mobilization dynamics. Higher plasma free fatty acids (FFA) were accompanied by lower blood insulin, leptin and glucose levels. After refeeding, a rebound in BW and BCS were observed, and FFA were drastically reduced in underfed ewes. No differences were detected in plasma FFA at the end of the study, but the lipolytic activity was different and contrasted with the adipose tissue mass.

## 1. Introduction

Maintaining the consistency of the internal environment (homeostasis) and/or sustaining productive functions (homeorhesis) are essential mechanisms for controlling metabolic processes, allowing animals to adapt to physiological and environmental perturbations [1]. How the animal partitions its nutrients when nutrients are limited, or imbalanced, is a major way in which it can cope with such variations and, thus, determines its robustness. In highly productive ruminants, there is evidence that their reliance on body energy reserves (BR) is increased, and body mass is reduced to fit significant energy requirements [2]. The efficiency of BR mobilization-accretion processes, to overcome undernutrition, is therefore recognized as an essential mechanism in ruminants [3,4,5,6]. These processes contribute to maintaining the resilience of the flock under a fluctuating feed supply, such as in tropical [7] or Mediterranean regions, where seasonal forage availability is highly fluctuating.

In previous works, we characterized the energy metabolism in a typical round productive year of Romane [8] and Lacaune [9] meat and dairy ewes, respectively, and the potential of plasma free fatty acids (FFA, formerly nonesterified fatty acids—NEFA) for being used as a predictor of the ruminant nutritional status was confirmed. Furthermore, we know that adipose tissue (AT) lipolytic potential can be estimated in vitro (by glycerol and FFA responses from tissue explants into the incubation medium) or in vivo by plasma glycerol or FFA response to an injection or infusion of catecholamines or synthetic drugs (β-adrenergic agonists) [10]. Such lipolytic potential could be seen as a sight of the ultimate necessity of the animal to meet their basic nutrient requirements by using their BR. When facing an undernutrition event, a quick BR mobilization (illustrated by increased plasma FFA) could be a symptom of the incapacity of the animal to readjust its maintenance energy requirements (MER), which would lead to regulating (reduce) its feed intake. Under the same conditions (i.e., species, breed, physiological state, age, production system, feeding regimen, etc.), less blood FFA in the immediate response would mean that the animal is less dependent on its BR in the very short term.

For this study, we hypothesized that offering restricted diets to adult *Merinos d’Arles* ewes would significantly increase their BR mobilization to meet their MER. After refeeding, the metabolic plasticity of this breed [11,12,13] would lead to recover the initial body condition within a similar period of time to that of the feed restriction. We also hypothesized that ewes with contrasting body condition scores (BCS), resulting from receiving different dietary regimes, would respond differently to an in vivo β-adrenergic challenge. That response will correspond to the individual reactivity or adaptive capacity.

Thus, the objective of this study was to evaluate the impact of offering diets of differing nutritional planes on the adaptive capacity of mature ewes at the short, medium and long terms. Such adaptations will be characterized by studying trends in the individual BR mobilization-accretions and the associated metabolic profiles after dietary challenges. A second objective was to evaluate the impact of different BCS on the individual lipolytic potential of the adipose tissue of the ewes facing a β-adrenergic challenge. This would allow us to study the potential of a simplified method for analyzing the intra-flock variability in individual metabolic plasticity responses when facing nutritional deficiencies.

## 2. Materials and Methods

### 2.1. Location

The experiment was conducted at the Montpellier SupAgro Domaine du Merle experimental farm, located in Salon-de-Provence in the Southeast of France (43°38′ N, 5°00′ E). All animals were cared in accordance with the guidelines of the *Institut National de Recherche pour l’Agriculture, l’Alimentation et l’Environnement* (INRAE) animal ethics committee. The experiment was approved by the INRAE and the Regional Ethic Group Montpellier (11/2015) and is compliant with the Animal Research Act 1985 in accordance with ethical principles that have their origins in the European Union directive 2010/63/EU.

### 2.2. Ewes, Management, Feeding and Experimental Design

After weaning their litters in mid-January, 36 6 to 10-year-old adult *Merinos d’Arles* ewes, which had lambed in October (average lambing on 10 October), were selected for this study from the main research flock. Body weight (BW) and BCS were used to select animals with similar body conditions. The initial BW and BCS were 44.4 ± 0.83 kg and 2.0 ± 0.05, respectively.

**Figure 1:**
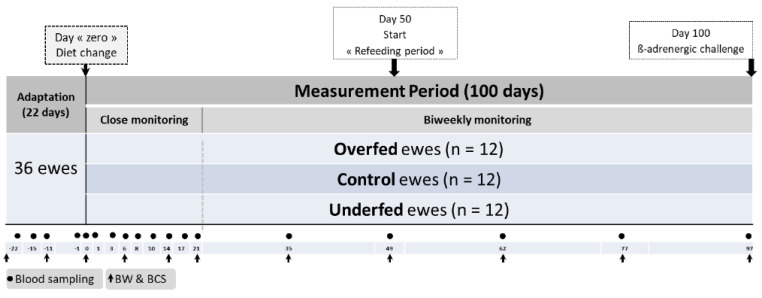
Schematic representation of the experimental design. The distribution of experimental ewes (*n* = 36) subjected to three planes of nutrition and the body weight (*n* = 11), blood (*n* = 18) sampling points and a final ß-adrenergic challenge is illustrated. After 3 weeks of adaptation, the energy diet content was changed in the two extreme experimental treatments (overfed and underfed). The measurement period (100 days) consisted of two periods i.e., a dietary challenge (from 0 to 49 days) and refeeding (from 50 to 100 days) periods. Sampling was structured in close (3 weeks) and a more extended (biweekly) individual monitoring periods. BW: body weight and BCS: body condition score.

The design of the experiment, which lasted 122 days and comprised two consecutive periods, is presented in Figure 1. First, ewes were allowed to accustom to the feeding regimen and environment of the facilities for 22 days (under confinement). All ewes were managed as a single flock and fed the same control diet (composition included below) throughout this period. They were fed ad libitum at 120% of their expected feed intake (i.e., allowing a 20% of feed refusals based on the previous days’ voluntary intake measurement). After adaptation, a measurement period of 100 days followed, beginning on day “zero”. Ewes were randomly assigned to one of three covered pens, each with an area of approximately 30 m^2^ and containing both concrete and straw flooring in the same sheep pen. The 100-day trial was divided into two periods (50 days each), including a dietary challenge period (from day 0 to 49) followed by a refeeding period (from day 50 to 100; Figure 1). On the last day of the trial, a ß-adrenergic challenge protocol was carried out (details included below).

Ewes were pen-fed, and feed was allocated according to treatment to the three diets in each of the roofed pens (*n* = 12 ewes/pen). The treatments included the following diets: (i) underfed ewes offered 70% of the theoretical MER (i.e., underfed diet), (ii) ewes offered 100% of the MER (i.e., control diet) and (iii) ewes offered 160% of the MER (i.e., overfed ewes or overfed diet). The decision for underfed and overfed rates were based, respectively, on criteria for an acceptable energy allowance restriction of 70% MER (i.e., without risking animal survival) and for easily allowing BR accretion (up to 160% of MER).

At the start of the experiment, ewes were on a maintenance diet. Considering the average BW (~45 kg BW; 17.4 kg BW^0.75^), the individual daily intake capacity was 1.3 sheep fill units (SFU). The rationing was carried out with the INRA feeding system [14]. One of the main particularities (before the new revised version of 2018 [15]) was its protein feeding system including a deep analysis of the protein truly digested in the small intestine (PDI) and the expression of the energy content of the feedstuffs as feeding units (UF), the net energy (NE) content of the particular feedstuff relative to that of the French reference barley (1.7 Mcal of NE/kg). The UF are expressed either in UF for maintenance and meat production (UFV) or for milk production (UFL). The protein content of the feedstuffs is expressed into two calculated PDI values, the PDIE and the PDIN, when rumen available energy or rumen available nitrogen are limiting for microbial growth, respectively. According to the INRA tables [14], to meet their MER, 0.033 SFU/kg BW^0.75^ for maintenance and meat production (UFV) and 2.3 g/kg BW^0.75^ of PDI was required. Therefore, feeding diets for each treatment were theoretically planned to achieve BCS of 1.75, 2.5 and 3.25 for underfed, control and overfed ewes, respectively.

The nutritive values of the ingredients included in the experimental diets are presented in Table 1. As the basal roughage, a wheat straw containing 3.5% of crude protein (CP), 1.34 Mcal/kg metabolizable energy (ME) dry matter (DM) basis and 2.4 UFV (INRA, 2010; [14]) was used. Dried and pelleted alfalfa (16% CP) was offered as the main protein source, whereas a dried and pelleted sugar beet pulp was supplied as the main energy source (2.7 Mcal/kg DM of ME and 1.0 SFU). A mineral-vitamin premix, containing 90 and 126-g/kg DM of P and Ca, respectively, was supplied at the same dose (~10 g/ewe/day) for all treatments (Table 2), thus ensuring the same amount of P and Ca (1 g/ewe/day). 

Table 2 presents the dietary composition for each experimental treatment, including the amounts of each ingredient and the overall daily nutrient supply (per ewe), according to each diet used in the study. The control diet was composed of 910 g of wheat straw, 165 g of alfalfa and 170 g dried sugar beet pulp. For the overfed diet, the quantities of alfalfa and sugar beet pulp were increased compared to the control, whereas the quantity of wheat straw was reduced. In contrast, underfed ewes were offered only 1 kg of wheat straw daily. These experimental diets corresponded to the dietary challenge period from day 0–49 following adaptation to it (Figure 1). During the second half of the trial (day 50 to the end of the experiment), an equivalent additional daily quantity (DM basis) of 100 g of alfalfa and 100 g of dried sugar beet pulp per ewe were supplied to each of the three experimental diets. During the refeeding period, wheat straw supplied to the underfed ewes was reduced to half of that supplied during the 0 to 49-day measurement period (Table 2).

Ewes were group-fed once daily at 08:00 h, and diets were provided ad libitum, which was weekly adjusted at 120% compared to the average intake for the previous week. Feed refusals were daily weighted, and samples were weekly pooled for further analyses. Ewes in each treatment had free access to fresh drinking water and mineral salts.

### 2.3. The ß-Adrenergic (Isoproterenol) Challenge

The dissimilar BCS attained among groups at the end of the experimental period allowed to induce a ß-adrenergic challenge to all ewes the last day. The objective of this method was to simulate a pronounced energy shortage challenge by injecting a β-adrenergic agonist, allowing to evaluate the lipolytic potential of the ewes’ AT. The kinetic of releasing plasma FFA, a predictor of undernutrition status in ruminants, is then compared with the 3 contrasted BCS groups attained with the experimental ewes after the nutritional challenge. 

The previous day of the β-adrenergic challenge, the ewes were individually weighed and the BCS estimated. All ewes were challenged early in the morning (~0800 h) at day 100. The challenge consisted on an intravenous injection (4 nmol/kg BW) of isoproterenol (ISO, Isuprel^TM^; Hospira France, 92360 Meudon-La-Fôret, France). Isuprel^TM^ (0.2-mg isoproterenol hydrochloride/mL sterile injection) is a potent nonselective β-adrenergic agonist with very low affinity for α-adrenergic receptors. For the individual monitoring of reactions, blood samples (*n* = 10) were drawn from each ewe by jugular venipuncture at −15, −5, 0, 5, 10, 15, 20, 30, 45 and 60 min relative to the β-adrenergic challenge time.

### 2.4. Measurements, Blood Sampling, Hormones and Metabolite Assays

Measurements were recorded for 122 days, starting with the acclimatization period (22 days) and continuing throughout the 100-day measurement period (Figure 1). Ewes were individually and manually monitored for BW (*n* = 11) and BCS [16] at 28 and 11 days before the experimental period (−28 and −11, respectively) and at days 0, 6, 14, 21, 35, 49, 62, 77 and 97 after the beginning of the dietary challenge. The ewes were weighed with an electronic static scale (Scale 300 kg UO1896, Société AGID, Dijon, France). The BCS was assessed according to an adaptation of the original grid described by Russel et al. [16], which was further divided into a 1/10 scale, i.e., from 1 to 5, with 0.1 increments. Similarly, plasma samples for the determination of metabolites and metabolic hormones associated with energy metabolism (*n* = 18) were taken at 22, 15, 11 and 1 days before the experimental period (−22, −15, −11 and −1, respectively) and at days 0, 1, 3, 6, 8, 10, 14, 17, 21, 35, 49, 62, 77 and 97 following the beginning of the dietary challenge.

The close monitoring of ewes (every two or three days) started the day before the dietary changes and lasted until 3 weeks after the beginning of the 100-day measurement period. Following this, approximately two sampling points per month were performed until the end of the experiment (Figure 1).

For monitoring the energy metabolism progression of ewes receiving each experimental diet, individual concentrations of plasma metabolites, including free fatty acids (FFA), beta-hydroxybutyrate (β-OHB), glucose (GLU), insulin (INS) and leptin (LEPT) were determined according to the protocols previously described [8,9]. Blood samples were taken by jugular venipuncture before the first meal (approximately at 0800 h) on each sampling day. Two 9-mL samples were drawn from each ewe (1 tube with 18 IU of lithium heparin per 1-mL blood and 1 tube with 1.2–2 mg of potassium EDTA per 1-mL blood; Vacuette^®^ Specimen Collection System, Greiner Bio-One GmbH, 4550 Kremsmünster, Austria). Samples were immediately placed on ice before centrifugation at 3600× *g* for 20 min at 4 °C. The plasma was collected and stored at −20 °C in individual identified aliquots (3 µL) for the metabolite and hormone analyses. Plasma FFA was measured in duplicate using the commercially available Wako NEFA-HR(2) R1 and R2 kit (manufactured by Wako Chemicals GmbH, Neuss, Germany and distributed by Laboratoires Sobioda SAS, Montbonnot, Saint Martin, France); intra- and inter-assay variations averaged 4.9% and 3.5%, respectively. Plasma GLU concentrations were measured in triplicate using a commercially available glucose GOD-PAP kit (reference LP87809; manufactured and distributed by Biolabo SAS, Maizy, France); intra- and inter-assay variations averaged 2.5% and 2.1%, respectively. Plasma β-OHB were measured in duplicate using the enzymatic method proposed by Williamson and Mellanby [17]; intra- and inter-assay variations averaged 8.8% and 3.3%, respectively. Plasma INS was measured in duplicate using a commercially available RIA kit (Insulin-CT; manufactured by MP Biomedicals, Solon, OH, USA and distributed by Cisbio Bioassays, Codolet, France); intra- and inter-assay variations averaged 10.3% and 4%, respectively. Plasma LEPT was quantified using the double-antibody leptin RIA procedures with some modifications, as previously described [8,9]; average intra- and inter-assay coefficients of variation were 5.4% and 4.8%, respectively.

For the ß-adrenergic challenge, the 9-mL blood samples (1 tube with 1.2–2 mg of potassium EDTA per 1-mL blood) drawn from each ewe at each sampling point of the kinetic (*n* = 10) were placed immediately on ice before centrifugation at 3600× *g* for 20 min at 4 °C. Plasma was harvested and stored at −20 °C until analyses in individual identified aliquots (3 µL). Concentrations of plasma FFA were analyzed in duplicate, similarly to the procedure previously described. Intra- and inter-assay variations for these samples averaged 4.74% and 6.98%, respectively.

### 2.5. Calculation and Statistical Analyses

Statistical analyses were performed using the Statistical Analysis System package (SAS; v. 9.1.3., 2002–2003 by SAS Institute Inc., Cary, NC, USA) [18]. Data were analyzed using the PROC MIXED function with repeated measures. Comparison of means was made using the least-squares means separation procedure of SAS using the PDIFF option according to the following statistical model:

*Yijk* = *µ* + *Diet_i_* + *Ewe_ij_* + *Time_k_* + (*Diet* × *Time*)*_ij_* + *ε_ijk_* (1)

where *Yijk* is the response at time *k* for ewe *j* that consumed the experimental diet *i*, *µ* is the overall mean, *Diet_i_* is the fixed effect of the specific experimental diet *i* (*i* = 1–3), *Ewe_ij_* is the random effect of ewe *j* offered the diet *i*, *Time_k_* is the fixed effect of time *k*, *(Diet × Time)_ik_* is the fixed interaction effect of the diet *i* for time *k* and *ε_ijk_* is the random error at time *k* on ewe *j* offered the diet *i*.

For the ß-adrenergic challenge database, the FFA response at each time after the challenge was calculated as the change in concentration from the basal (−15 min) value, as described by Chilliard et al. [19]. The area under the concentration curve (AUC) was calculated by doing a definite integral between the two points or limits at time X using the following formula:

AUC1-2 = (B1 + B2)/2 × (A2 − A1)(2)

where B is the y axis value (FFA concentration) and A is the *X*-axis value (time relative to challenge). The AUC was thus calculated for each ewe for the time intervals 0 to 5 min (AUC05), 5 to 10 min (AUC510), 10 to 15 min (AUC1015), 15 to 30 min (AUC1530), 30 to 60 min (AUC3060) and, finally, from 0 to 60 min (AUC060).

By using the data from the concentration-time plot, we calculated the FFA elimination rate (turnover) constant of each ewe after the ISO challenge (i.e., rate at which FFA was cleared from the body). For this purpose, we calculated K, which is the slope of the regression line between time (hours). The measured concentration values of FFA above the initial point (t = 0; time of injection of ISO) was firstly transformed to their natural logarithm. Extrapolation at zero time gives the theoretical maximal amplitude above the initial point (FFAamp). Since we did not have access to the volume diffusion or volume of distribution (V) of FFA, we used the individual BW of each ewe to determine the clearance rate, which was calculated as follows:

CL = K × BW(3)

where CL is the FFA clearance rate value from the body of each ewe, K is the slope of the regression line and BW is the individual BW at the adrenergic challenge moment.

Data of FFA kinetics in the β-adrenergic challenge were analyzed as repeated measures ANOVA using the PROC MIXED function with the least-squares means separation procedure using the PDIFF option of SAS. The statistical model was as follows:

*Y_ijk_* = *µ* + *BCS_i_* + *Ewe_ij_* + *Timek* + (*BCS* × *Time*)*_ij_* + *ε_ijk_* (4)

where *Y_ijk_* is the response at time *k* on ewe *j* with a *BCS_i_*, *µ* is the overall mean, *BCS_i_* is a fixed effect of the BCS of the ewe at the moment of challenge *i* (*i* = 1–3), *Ewe_ij_* is a random effect of ewe *j* with a *BCS_i_*, *Time_k_* is a fixed effect of time relative to challenge *k*, *(BCS × Time)_ik_* is a fixed interaction effect of the BCS of the ewe *i* with time relative to challenge *k* and *ε_ijk_* is random error at time *k* on ewe *j* with a *BCS_i_*.

The BW, BCS and FFA responses after the challenge concerning basal FFA and AUC at different periods were analyzed by the ANOVA procedure of SAS considering the fixed effect of the BCS group of the observation value. Results were considered significant if *p* < 0.05. Correlation coefficients between basal plasma FFA and plasma FFA responses to the ISO challenge and AUC at different ranges of time and from time 0 to 60 min were determined using the PROC CORR of SAS.

## 3. Results

**Figure 2:**
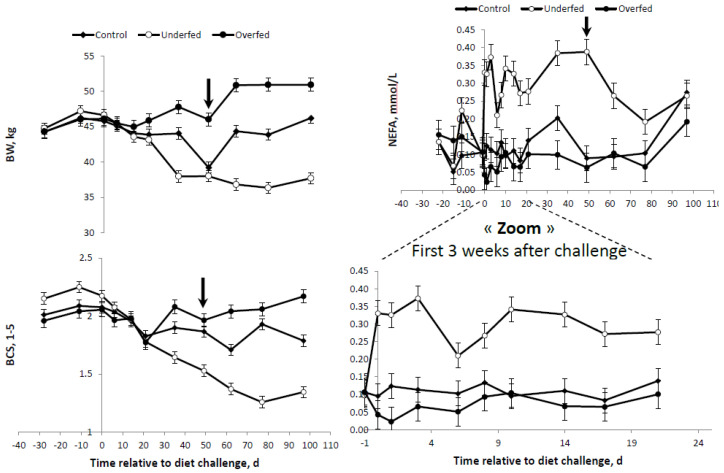
Body weight (BW), body condition score (BCS) and plasma free fatty acids (FFA or nonesterified (NEFA) concentrations of mature, dry, nonpregnant *Merinos d’Arles* ewes (*n* = 36) offered 70% (underfed; *n* = 12), 100% (control; *n* = 12) or 160% (overfed; *n* = 12) of maintenance energy requirements. Diet challenge started at day 0, after a 3-week adaptation period. Arrow represents commencement of refeeding period. Bars are SEM.

The final average individual daily feed balance of the sheep is shown in Table 3. After calculating the average feed refusal per treatment for each stage of the measurement period (dietary challenge period from 0–49 days and refeeding period from 50–100 days), it was determined that ewes were 114%, 68% and 190% of their MER for the control, underfed and over groups, respectively. This was different from the 100%, 70% and 160% MER theoretically planned, respectively (Table 2). However, the final objective of MER for each of the experimental diet treatments was attained. Overall changes in BW, BCS and plasma profiles are presented in Table 4. When all variables were considered, significant effects were observed for the main sources of variation evaluated, i.e., the diet, time after diet challenge and their first order interactions. A significant effect was observed for the interaction diet × time for all variables measured. As expected, after beginning the feeding regimen (day “zero”), the average BW and BCS were higher and lower in the overfed and underfed treatments (Table 4 and Figure 2). At the beginning of the refeeding period (day 50), a significant recovery of BW and BCS was observed.

The differences and trends observed for BW and BCS were consistent with those obtained for plasma FFA concentrations (Figure 2). The higher and lower average BR mobilization, as illustrated by the plasma FFA concentration, were observed in the underfed and overfed ewes. However, the differences between the overfed and control diets were only evident until one week following the change of the diet (Figure 2). Once the refeeding period started, plasma FFA were drastically reduced in underfed ewes. At the end of the study, no significant differences were detected between the treatments (Figure 2).

**Figure 3:**
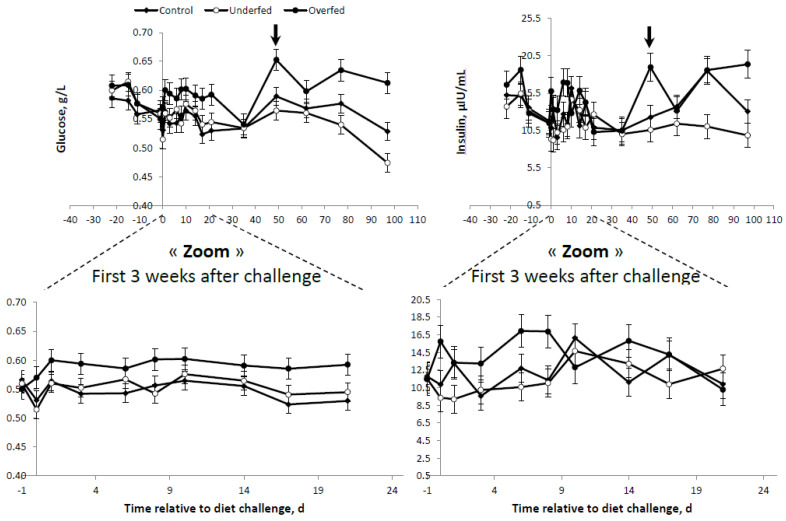
Plasma glucose and insulin concentrations of mature, dry, nonpregnant *Merinos d’Arles* ewes (*n* = 36) offered different diets, i.e., 70% (underfed; *n* = 12), 100% (control; *n* = 12) or 160% (overfed; *n* = 12) of maintenance energy requirements. Diet challenge started at day 0, after a 3-week adaptation period. Arrow represents commencement of refeeding period. Bars are SEM.

Differences in plasma GLU were only observed between overfed ewes and the other treatments, with no differences between underfed and control ewes (Table 4 and Figure 3). Conversely, plasma INS concentrations were more consistent with the differences in the BR mobilization rates (i.e., FFA profiles) shown above. The higher the plasma FFA concentrations, the lower the INS concentration. Therefore, plasma INS was higher in the overfed ewes, followed by the control ewes, and was lowest for underfed ewes (Table 4 and Figure 2 and Figure 3). A concomitant, parallel effect on INS and GLU was observed following refeeding. The peak in the GLU concentration observed in the overfed ewes was followed by a similar peak for the plasma INS profile at the same time point and in the same group of ewes. In general, either plasma GLU or INS concentrations were higher in the overfed ewes throughout the experiment, but no differences existed in control and underfed ewes (Figure 3). 

**Figure 4:**
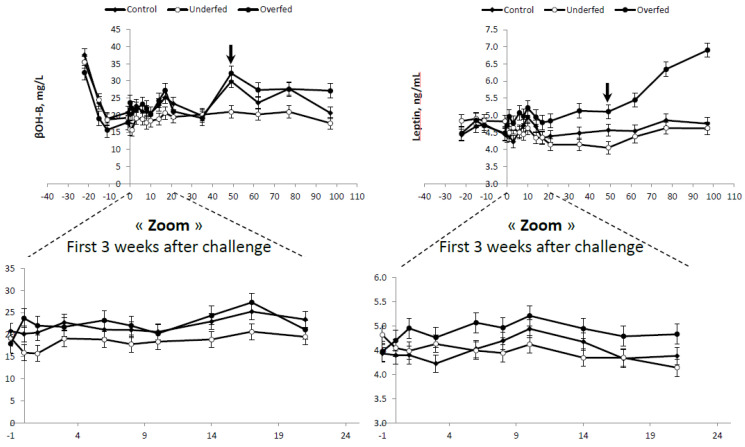
Energy metabolism (β-hydroxybutyrate—β-OHB—and leptin) plasma profile of mature, dry, nonpregnant *Merinos d’Arles* ewes (*n* = 36) offered different diets during the dry-off period, i.e., 70% (underfed; *n* = 12), 100% (control; *n* = 12) or 160% (overfed; *n* = 12) of the maintenance energy requirements. Diet challenge started at day 0, after a 3-week adaptation period. Arrow represents commencement of the refeeding period. Bars are SEM.

No differences in plasma β-OHB concentrations were found between control and overfed ewes (Table 4). Lower (*p* < 0.003) plasma β-OHB concentrations were observed in the underfed ewes compared to the average of the control and overfed ewes combined (20.16 ± 0.684 vs. 23.47 ± 0.876 mg/L, respectively). The lower plasma β-OHB profile observed in underfed ewes was consistent throughout the experiment (Figure 4).

Differences in plasma LEPT were also consistent with the feeding regimen (overfed > control > underfed; Table 4). As expected, higher plasma LEPT concentrations were observed in the overfed group throughout the experimental period, and these differences increased (*p* < 0.01) following refeeding (Table 4 and Figure 4).

**Figure 5:**
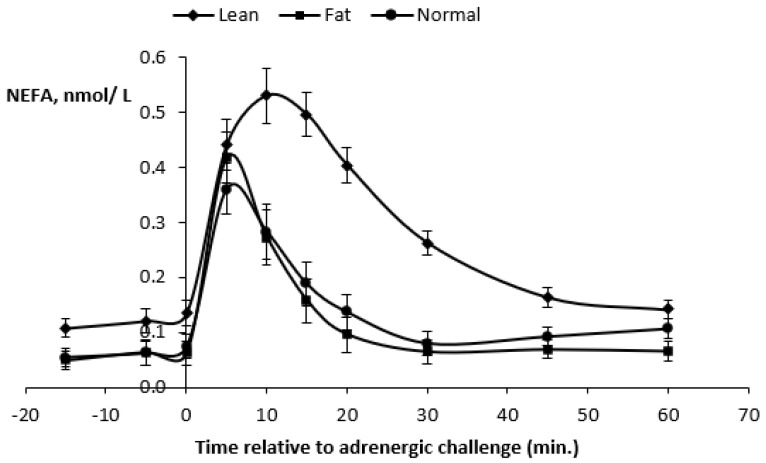
Effects of the β-adrenergic challenge injection with isoproterenol (ISO, 4 nmol/kg BW) on plasma nonesterified (NEFA) or free fatty acids (FFA) kinetics of mature, dry, nonpregnant *Merinos d’Arles* ewes (*n* = 36) with low (underfed or lean ewes), medium (control or normal ewes) and high (overfed or fat ewes) body condition scores. Bars are SEM.

At the ß-adrenergic challenge, significant differences (*p* < 0.0001) were detected when comparing the average BW and BCS of the three experimental treatments (Table 5 and Figure 5). As a result of the previous 100-day feeding period, underfed ewes (BW = 37.7 kg and BCS = 1.34) were more than 10 kg lighter than control ewes (BCS = 1.79) and overfed ewes (BCS = 2.17) ewes (46.2 and 50.9 kg BW, respectively).

Plasma FFA (−15 min) before the β-adrenergic challenge were higher (*p* < 0.0002) in underfed ewes compared to control and overfed ewes (Table 5). In contrast, plasma FFA responses at 5 min after the ISO challenge were higher (*p* < 0.0003) in overfed ewes. After 10 min, plasma FFA responses were consistently higher in underfed ewes. Plasma FFA maximal responses (0.56 mmol/L) were higher (*p* < 0.0001) in underfed ewes, and this occurred at 12 min after the challenge (Table 5 and Figure 5).

The AUC were all higher (*p* < 0.0001) in underfed ewes. Thus, the overall results showed that underfeeding increased the basal plasma FFA, plasma FFA response at 10 and 20 min, plasma FFA maximal response after the ISO challenge and all FFA response areas (*p* < 0.0001). The FFA maximal response occurred later in underfed ewes when compared to control and overfed ewes.

Plasma FFA kinetics for the three treatments are presented in Figure 5. Plasma FFA concentrations increased for 10 min in all ewes and were always higher (*p* < 0.0001) in underfed ewes during the 60 min post-challenge, with a peak plasma FFA concentration attaining 0.53 mmol/L. However, for all ewes, the plasma FFA concentrations decreased similarly, and, after 60 min, they returned to values close to the baseline.

All correlations (*r* = 0.54 to 0.79) between the basal FFA and different variables of the FFA responses to the ISO challenge were significant (*p* < 0.0001; Table 6), except the variable time between the ISO challenge and maximal response (*r* = 0.25). The highest correlations between the basal plasma FFA and responses at different points after the challenge were at 10 or 15 min (*r* = 0.73 and 0.69, respectively). The highest correlation between the plasma FFA and AUC was in the area from 0 to 15 min.

From 20 min after the challenge, the correlations were progressively lower in the sense of the declining tendency of the curve. Correlations with AUCs from time 0 to 60 min were very high for responses at 10, 15, 20 min and the maximal response. Correlations between the AUC from time 0 to 60 min and the AUC from time 10 to 15 min, or the AUC from time 15 to 30 min were very strong (*r* = 0.97); correlations between the AUC from time 0 to 60 min and the AUC from time 30 to 60 min (declining part of the curve) were also high (Table 6). Correlations between the maximal response and the response at 5 or 10 min and with the AUC from time 5 to 10 min were high (ranging from 0.83 to 0.89; Table 6).

**Figure 6:**
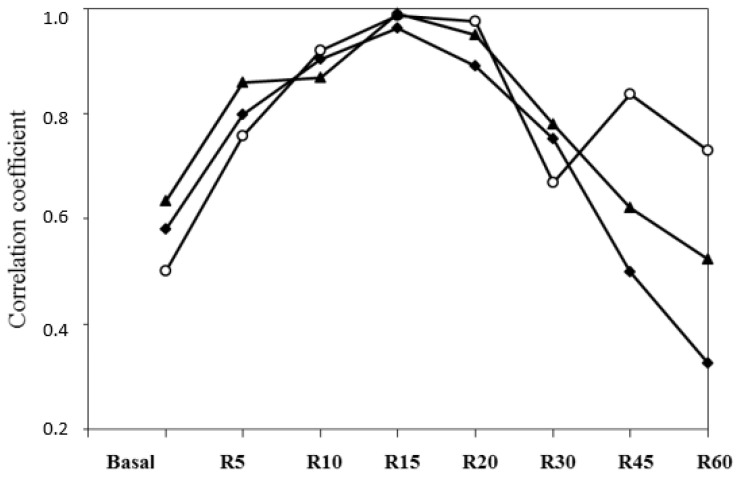
Correlations between the plasma area under the concentration curves (AUC) from time 0 to 60 min and basal plasma FFA or plasma FFA responses (R) at different times after the isoproterenol challenge in control (-o-), underfed (black-filled triangle) or overfed (fat, black-filled diamond) *Merinos d’Arles* meat ewes.

Within BCS diet correlations between the AUC from time 0 to 60 min and responses at 10, 15 and 20 min, they were higher than those with basal plasma FFA or FFA responses at 5, 30, 45 and 60 min (Figure 6). Differences in the releasing FFA turnover were observed between ewes and among individuals in the same treatment and with similar BW and BCS statuses.

## 4. Discussion

In the future, the sustainability of farming systems will rely on their ability to cope with a reduction of external inputs such as concentrate and other feed resources. In this context, a better understanding of the relationship between the nutrients supply, nutritional status, their interactions with BR dynamics and the progression of the metabolic profile is essential for the development of more comprehensive nutrition managements of animals based on their relative adaptive capacities [20,21,22,23,24]. The objectives of this study were to evaluate and describe how dietary energy restrictions and/or repletion influences change in BW, BCS and metabolic status responses in *Merinos d’Arles* ewes, considered to be a robust, rustic and hardy sheep breed. It is noteworthy to highlight that we focused on the energy density of the diets; however, one may expect that experimental treatments also induced different protein balances between the diets, which were also likely affecting, even if indirectly, the overall animal responses. Our approach is different (and complementary) to that used by other authors more focused in mineral and protein metabolism mechanisms (including nutrient uptake and mobilization in muscle and hepatic tissues) explaining sheep’s ability to withstand weight loss [23,24].

We validated the previous estimation of the energy requirements (INRA, 2010 [14]) of the dry ewe by stabilizing the BCS and BW over the whole experimental period with the control diet. Hence, the adaptive capacity of the underfed and overfed ewes will be discussed by direct comparison with the control ewes’ responses. We confirmed that offering diets restricted in nutrients to ewes would induce significant increases in BR mobilization to meet their energy and protein requirements. We also verified that, after partial refeeding, the metabolic plasticity of these ewes allowed recovering of the BW and BCS within a period of similar duration to that of the feed restriction, whereas a high restriction of nutrient allowances would temporarily and negatively affect the feed intake. The highest feed refusal rate was consistently observed in the underfed ewes, mainly after the first three to four weeks of feeding the restricted diet. This is likely to be a consequence of a depressed ruminal environment together with low roughage quality, which is known to negatively affect digestion, metabolism and appetite in underfed ruminants.

Differences in the BW and BCS progression throughout the experiment were expected, considering the dietary energy manipulation. The underfed, control and overfed ewes reduced, maintained and improved their BW and BCS, respectively. Underfed and control ewes responded to the dietary manipulation by maintaining their BW and BCS, as observed with the reduced plasma GLU, INS and LEPT concentrations, compared to overfed ewes.

Throughout the experiment, underfed ewes (who consumed almost half of their MER) presented lower BW and BCS, increased plasma FFA concentrations and lowered plasma INS and LEPT concentrations when compared to adequately fed ewes. The BR mobilization status was well-illustrated by the consistently higher plasma FFA concentrations in underfed ewes. This was expected, as this treatment was exposed to a severe energy restriction, based on only wheat straw for the first 50 days of the measurement period. After beginning the refeeding period, underfed ewes responded to the energy repletion with an immediate short-term decrease in their plasma FFA concentrations, with a more delayed recovery of the BW and BCS, which made sense, since it was only a partial refeeding.

The endocrine system, characterized by plasma INS and LEPT profiles in this study, regulates metabolism by finely tuned peripheral information, which are ultimately aimed to maintain homeostasis. These adaptive processes involve the interplay between several hormones, such as growth hormone (GH) and insulin-like growth factors (IGF-1) [25,26,27,28], which were not analyzed in this study.

The typical characteristics of underfed ruminants were observed in the underfed ewes. The lack of glucose arriving to the rumen, in addition to a reduction in volatile fatty acids (VFA) production, induced gluconeogenesis accompanied by intense lipolysis, proteolysis and ketogenesis [1,25]. The reduction of the gut metabolic activity is known to account for the decrease of the energy requirement in underfed ruminants. Thus, the reduced oxidative or basal metabolism is characterized by a decrease in plasma GLU, INS, LEPT and prolactin concentrations and an increase in other hormones, such as GH, adrenalin, cortisol and glucagon. This generally leads to shifts in the metabolic pathways that aim to spare GLU (with the accompanied increase in FFA) and proteins (with increased proteolysis and ketogenesis). The enhanced gluconeogenesis role in underfed ewes probably explained the lack of differences in the plasma GLU of these ewes when compared to the control ewes.

Interestingly, the plasma β-OHB level was lowest in ewes receiving the underfed diet. Considering that β-OHB is the resultant of BR mobilization but, also, butyrate availability from ruminal digestion, we speculate that lower plasma β-OHB concentrations in underfed ewes may be due to limits in the supply of β-OHB precursors in their diet. Thus, even if a higher BR mobilization was present in underfed ewes, the lower β-OHB plasma concentration is likely the consequence of the ingredients used in the experimental diets. The underfed diet, which only offered wheat straw, did not contain the required precursors for producing this metabolite—for example, single sugars from molasses to enhance the ruminal butyrate production that will further actively participate in the β-OHB found in plasma.

Underfed fat-tailed Barbarine ewes were reported to be able to produce and survive thanks to their significant ability to mobilize their BR [29]. Plasma FFA and β-OHB concentrations were initially almost doubled. The medium-term responses of these ewes were very similar to what we observed: a steady decline of these blood metabolites, which was attributed to their ability to adjust their lipid metabolism to reduce the toxic effects of high plasma FFA and β-OHB concentrations and, therefore, prolong survival. After partial refeeding, Barbarine ewes were able to fully recover their initial BW, lipid and protein masses [29].

Subjected to overfeeding, the enhancement of the anabolic pathway response was clear in the overfed ewes, with increased plasma GLU, INS and LEPT concentrations and decreased plasma FFA. However, differences toward the dominant anabolic responses were not always evident when comparing the control and overfed ewes, despite the clear differences in energy supply, which should have been sufficient to create greater differences than those observed. However, BW and BCS progressions were different between the experimental group of ewes from 20 days after the diet changes. Thus, differences in the responses between these two diets were not well-expressed in this study from the analysis of the chosen blood metabolites and metabolic hormones. This led us to hypothesize that measuring other variables, including GH, IGF-1 and plasma urea nitrogen (PUN), may improve the characterization of the anabolic and catabolic responses. It is highly probable, as previously reported [30], that increasing the amounts of stored body fat in the overfed ewes increased their MER, thus reducing the energy balance gaps between the control and overfed ewes. Hence, metabolic and endocrine profiles are blunted by this phenomenon. Such effects of body fatness on energy requirements were reported in underfed fat ewes [28]. Ewes with low BCS in that study had lower plasma GLU, triiodothyronine and thyroxine concentrations and serum INS, albumin, globulins and IGF-I, in addition to higher serum FFA, urea and creatinine.

When the energy intake is high, blood INS concentrations are also high, which promotes growth and/or BR recovery [1,27,30]. Such a positive correlation between energy intake and blood INS concentrations has been reported, and this response was confirmed as the lowering energy supply decreased the plasma INS concentrations during energy restriction [26].

### 4.1. Adrenergic Challenge

Our results regarding the responses to the ß-adrenergic challenge are in agreement with Chilliard et al. [19]. These authors found that basal FFA and FFA responses to an isoproterenol (ISO) challenge with a similar dose to that used in our experiment were higher in underfed than overfed cows. Consistent with our findings, high correlations between the response area or maximal FFA response and FFA response at 15 min (*r* = 0.95 and 0.98, respectively) were observed. A significant effect of BCS on the basal plasma level was also reported, thus indicating that the blood FFA response to ISO at 15 min could provide an efficient method for studying in vivo the AT lipolytic potential. Our results, including trends of the response curves with regard to BCS diets, are very similar to those findings, except the maximum value was obtained at 10 min in the present study. We also agree with the fact that the maximal response occurred later when this response was higher, which illustrates that the lipolytic response to ISO takes longer in underfed animals.

The strong correlation (*r* = 0.69) between the plasma FFA and FFA maximal response to ISO confirms the results obtained in lactating ewes [31] and suggests that the adrenergic component of the lipolytic cascade plays a significant role in the regulation of the basal plasma FFA. The plasma FFA response to the ISO challenge in well-fed lactating ewes depended on the body lipid mass but not on the energy balance. On the contrary, in underfed ewes, the FFA response depended on the energy balance and not on the body lipid mass. The adrenergic challenge was also useful in explaining the differences in interindividual adaptive strategies to underfeeding in the ewes. In underfed ewes, it has been observed that the relative variation in milk yields was negatively correlated to FFA+10 (*r* = −0.51), which show that the ability to support lactation was related to the ability to mobilize body lipids [31].

### 4.2. Potential Contribution for a Simplified Method Helping to Identify Individual Adaptive Capacities or Robustness (Intraflock Variability)

There is evidence of the great potential of plasma FFA as a powerful predictor of the nutritional status of ruminants under determined circumstances. This variable provides reliable information on the stage of the BR mobilization of the animal under a pressing physiological status and/or when facing the consequences of being reared in fluctuating environments [3,4]. Blood FFA is thus recommended as a good diagnostic tool for health or reproductive interpretations, and we think that it could also be considered as a pertinent marker to be included in models aiming to analyze the metabolic plasticity of ruminants when facing underfeeding in a given timespan (i.e., individual robustness).

In previous works carried out by our team and aiming to characterize the energy metabolism of ewes in a typical round productive year, the blood FFA potential for predicting the BR status was confirmed in both Romane [8] and Lacaune [9] ewes. In the present study, we evaluated the in vivo method with a β-adrenergic challenge. We confirmed our hypothesis that ewes with divergent BCS would respond differently to a β-adrenergic challenge and that this response could be predicted at a given point (10 min) of the plasma FFA kinetic after the challenge, in the function of the relationships between the different variable responses at different times.

Chilliard et al. [19], using the same method, looked for a simplified procedure for predicting the lipolytic response curve with a smaller number of samples after the ISO challenge. Consistent with our results, they obtained a very good prediction using the AUC from time 0 to 60 min, either by the partial AUC from time 0 to 20 min (*r* = 0.95) or by the sole response at 15 (*r* = 0.95) or 20 min (*r* = 0.97). Measuring the plasma FFA just before and at 15 (or 20) min after an ISO injection was thus considered as an efficient and simple way of predicting the maximal FFA response of a meat ewe and an AUC equivalent to one hour of sampling. Extra blood samplings at 5, 10, and 20 (or 15) min after the ISO challenge only slightly increased the prediction of these parameters.

The adaptive capacity of a ruminant to an adrenergic challenge (i.e., pronounced energy shortage) is expressed by subsequent individual physiological responses at the short-, medium- and long-term timespans. Differences in the amplitude (gap between maximum FFA response and basal FFA), turnover (exponential slope when reducing plasma FFA after maximal response) and length of those specific and combined processes are expected to be consistent with adaptive capacities’ differences between individuals reared under similar conditions.

**Figure 7:**
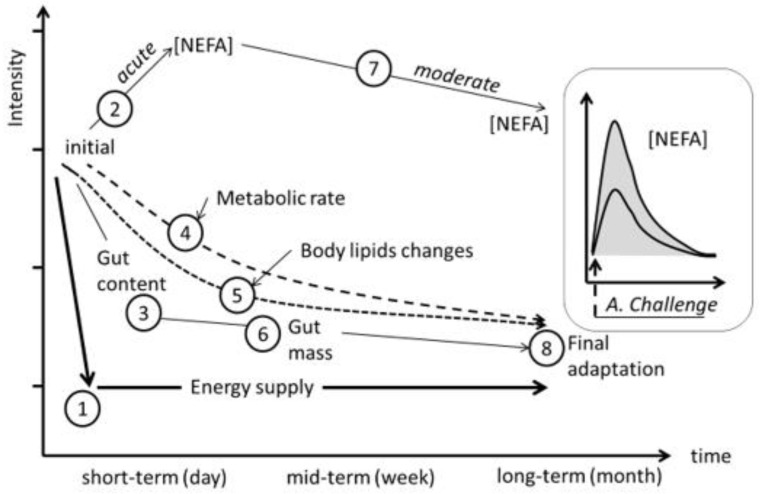
Schematic representation of the encompassing chain of short-, medium- and long-term reactions of a ruminant when facing a drastic feed (energy) shortage. NEFA = nonesterified or free fatty acids.

A stronger lipolytic potential could be seen as a sight of the ultimate necessity of the animal to compensate their basic requirements by mainly using their BR. Indeed, when facing undernutrition (i.e., challenge), a higher and quicker BR mobilization peak (illustrated by plasma FFA) could be a symptom of the incapacity of the animal to readjust its MER in the short term (Figure 7). This would be in close relationship with their more or less efficient capacity of regulating (reducing) their feed intake and, thus, their individual MER, which would mean a higher reliance on their BR *per se* to satisfy their energy requirements. Thus, under uniform conditions (i.e., same species, breed, physiological state, age, production system and feeding regime) less plasma FFA at a given point after the challenge would means that the animal is less reliant on its BR. Individuals with more pronounced blood FFA amplitudes and quicker FFA turnovers would be, a priori, better adapted animals when compared to their cohorts with less FFA amplitudes and slower FFA turnovers. Such differences at the intragroup level were observed in our experiment (Figure 7). This could enable us to the potential effective uses of this relative easy and quick method for acquiring useful information for identifying existing intraflock variability in individual robustness in practice at a given field situation, which is a relevant field of research in the current context of climate change [32,33].

## 5. Conclusions

These findings confirmed the ability of these mature, dry, nonpregnant *Merinos d’Arles* ewes to quickly overcome undernutrition situations by efficiently using their body reserves. The anabolic or catabolic responses to energy dietary manipulations were accompanied by synchronized metabolic regulation, resulting in differences in their metabolic and BCS profiles.

Since the lipolytic activity of the adipose tissue differed among ewes with similar body condition statuses receiving the same diets, our results also indicate the potential of using a simplified ß-adrenergic challenge protocol for identifying, at the intraflock level, individual differences in their adaptive capacity to undernutrition.

Our work contributes new knowledge to continue advancing in the subject of the individual robustness of ruminants subjected to the more and more frequent undernutrition situations in the current context of climate change. This is particularly relevant for sheep and small ruminants’ production systems under Mediterranean and tropical conditions.

## Figures and Tables

**Table 1:** Nutritive value of ingredients included in the experimental diets.

Ingredient	^1^ DM, %	Organic Constituents, g/kg DM			Energy, Mcal/kg DM		Minerals, g/kg DM			Net Energy, /kg DM	Protein Value, g/kg DM			Fill Value
		^2^ OM	^3^ CP	^4^ CF	^5^ GE	^6^ ME	Ash	P	Ca	^7^ UFV	^8^ PDIA	^9^ PDIN	^10^ PDIE	^11^ SFU
Wheat straw	88	920	35	420	4.34	1.34	80	1	2	0.31	11	22	44	2.41
Alfalfa (pelleted)	91	885	160	310	4.40	1.84	115	-	-	0.56	50	101	87	0.80
Dried sugar beet pulp	89	912	98	206	4.01	2.73	88	1	13	0.99	40	63	106	1.36
Mineral-vitamin premix	90	-	-	-	-	-	-	90	126	-	-	-	-	-

^1^ DM = dry matter content, ^2^ OM = organic matter content, ^3^ CP = crude protein content, ^4^ CF = crude fiber content, ^5^ GE = gross energy, ^6^ ME = metabolizable energy, ^7^ UFV = net energy for maintenance and meat production, ^8^ PDIA = dietary protein undegraded in the rumen, which is digestible in the small intestine, ^9^ PDIN = PDIA + PDIMN (microbial protein that could be synthesized from the rumen degraded dietary N when energy is not limiting), ^10^ PDIE = PDIA + PDIME (microbial protein that could be synthesized from the energy available in the rumen when degraded N is not limiting) and ^11^ SFU = fill unit for sheep.

**Table 2:** Diet composition and daily nutrient supply (per ewe), according to the diet (treatment) applied during the challenge period (from day “zero” to 49). Values between brackets correspond to the added or reduced quantities (in percentage with regard to the “diet challenge” period) applied during the refeeding period (from day 49 till 100).

Treatment (Diet)	Diet ingredient	Nutrient Supply (DM Basis)								
		Distributed (As-Fed), kg	DM, kg	^1^ UFV	^2^ PDIN, g	^3^ PDIE, g	SFU	^4^ CP, g	P, g	Ca, g
Control (100% ^6^ MER)	Wheat straw	0.91 (=)	0.80 (=)	0.25 (=)	18 (=)	35 (=)	1.28 (=)	28 (=)	1 (=)	2 (=)
	Alfalfa	0.17 (+11)	0.15 (+0.10)	0.08 (+0.06)	15 (+10)	13 (+9)	0.12 (+0.8)	24 (+16)	1 (+0.5)	1 (+0.5)
	Dried sugar beet pulp	0.17 (+11)	0.15 (+0.10)	0.15 (+0.10)	9 (+7)	16 (+11)	0.12 (+0.8)	15 (+10)	0 (+0.3)	2
Underfed (70% MER)	Wheat straw	1.00 (−0.50)	0.88 (−0.38)	0.27 (−0.11)	19 (−8)	39 (−17)	1.41 (−0.61)	31 (−13)	1 (=)	2
	Alfalfa	0 (+10)	0 (+0.10)	0 (+0.06)	0 (+10)	0 (+9)	0 (+0.08)	0 (+16)	0 (=)	0 (=)
	Dried sugar beet pulp	0 (+11)	0 (+0.10)	0 (+0.10)	0 (+6)	0 (+11)	0 (+0.08)	0 (+10)	0 (=)	0 (+1)
Overfed (160% MER)	Wheat straw	0.57 (+11)	0.50 (=)	0.16 (=)	11 (=)	22 (=)	0.80 (=)	18 (=)	1 (=)	1 (=)
	Alfalfa	0.44 (+10)	0.40 (+0.10)	0.22 (+0.06)	40 (+11)	35 (+9)	0.32 (+0.08)	64 (+16)	1 (+0.5)	1 (+0.5)
	Dried sugar beet pulp	0.45 (+11)	0.40 (+0.10)	0.40 (+0.10)	25 (+7)	42 (+11)	0.32 (+0.08)	39 (+10)	0 (+0.5)	5 (+1.5)

^1^ UFV = net energy for maintenance and meat production, ^2^ PDIN = PDIA + PDIMN (microbial protein that could be synthesized from the rumen degraded dietary N when energy is not limiting), ^3^ PDIE = PDIA + PDIME (microbial protein that could be synthesized from the energy available in the rumen when degraded N is not limiting), ^4^ CP = crude protein and ^6^ MER = maintenance energy requirements. The mineral-vitamin premix was supplied at the same rate for all treatments, i.e., 10 g/ewe/d, thus providing the same amount of P and Ca (1 g/ewe/d and 1 g/ewe/d, respectively).

**Table 3:** Final average individual daily feeding balance, after calculating the average determined feed refusal per dietary treatment for each stage of the measurement period (i.e., challenge, from 0 to 49 days, and refeeding, from 50 to 100 days).

Treatment	^1^ UFV					^2^ PDIN				
	Requirements	Actual Daily Intake			Balance, % of ^3^ MER	Requirements	Actual Daily Intake			Balance, % of ^4^ MPR
		0–49 d	50–100 d	Average			0–49 d	50–100 d	Average	
Control	0.592	0.63	0.72	0.675	114	41	47	60	53	129
Underfed	0.592	0.41	0.39	0.403	68	41	22	30	26	62
Overfed	0.592	1.09	1.16	1.123	190	41	102	107	104	252

^1^ UFV = net energy for maintenance and meat production, ^2^ PDIN = PDIA + PDIMN (microbial protein that could be synthesized from the rumen degraded dietary N when energy is not limiting), ^3^ MER = maintenance energy requirements and ^4^ MPR = maintenance protein requirements.

**Table 4:** Average body weight (BW), body condition score (BCS) and plasma metabolites and hormone concentrations of mature, dry, nonpregnant *Merinos d’Arles* ewes (*n* = 36) receiving 70% (underfed; *n* = 12), 100% (control; *n* = 12) or 160% (overfed; *n* = 12) of their maintenance energy requirements during the dry-off period. Values are least-squares (LS) means ± SEM. FFA: free fatty acids and β-OHB: beta-hydroxybutyrate.

Item	Experimental Diets			Effects, *p*-Value		
	Control	Underfed	Overfed	Diet	Time	Diet × Time
BW, kg	44.27 (±0.285) ^b^	41.59 (±0.285) ^a^	47.48 (±0.446) ^c^	<0.0001	<0.0001	<0.0001
BCS, 1–5	1.92 (±0.017) ^b^	1.78 (±0.017) ^a^	2.00 (±0.027) ^c^	<0.0001	<0.0001	<0.0001
FFA, mmol/L	0.12 (±0.011) ^b^	0.26 (±0.011) ^c^	0.09 (±0.017) ^a^	<0.0001	0.0002	<0.0001
β-OHB, mg/L	23.36 (±0.684) ^b^	20.16 (±0.684) ^a^	23.58 (±1.068) ^b^	0.003	<0.0001	<0.0001
Glucose, g/L	0.55 (±0.005) ^a^	0.55 (±0.005) ^a^	0.59 (±0.008) ^b^	0.025	<0.0001	0.0061
Insulin, µIU/mL	12.98 (±0.520) ^b^	11.58 (±0.520) ^a^	14.96 (±0.813) ^c^	0.004	0.0033	0.0001
Leptin, ng/mL	4.56 (±0.058) ^a^	4.52 (±0.058) ^a^	5.09 (±0.091) ^b^	0.003	<0.0001	<0.0001

^a–c^ Means bearing different superscript letters differ significantly (*p* < 0.05).

**Table 5:** Average body weight (BW), body condition score (BCS), plasma FFA (at −15 min) and plasma FFA responses to a β-adrenergic challenge with an isoproterenol injection in mature, dry, nonpregnant *Merinos d’Arles* ewes (*n* = 36) with different body condition scores.

Item	BCS Treatment						Effect, *p*<
	Control		Underfed		Overfed		
	LS Means	SEM	LS Means	SEM	LS Means	SEM	
Body weight, kg	46.22	0.786	37.69	0.786	50.92	0.923	<0.0001
Body condition, 1–5	1.79	0.049	1.34	0.049	2.17	0.058	<0.0001
Basal FFA, mmol/L	0.06	0.02	0.11	0.02	0.05	0.02	0.0002
Response at 5 min, mmol/L	0.31	0.04	0.33	0.04	0.37	0.04	0.0003
Response at 10 min, mmol/L	0.23	0.04	0.42	0.04	0.22	0.04	<0.0001
Response at 20 min, mmol/L	0.08	0.03	0.30	0.03	0.05	0.03	<0.0001
Maximal response, mmol/L	0.38	0.04	0.56	0.06	0.42	0.03	<0.0001
Time ^¶^, min	5.83	0.54	12.00	1.93	5.00	0.00	<0.0001
AUC ^Ø^, mmol.min/L							
from 0 to 5 min	1.1	0.14	1.4	0.23	1.2	0.09	<0.0001
from 5 to 10 min	1.6	0.22	2.4	0.33	1.7	0.14	<0.0001
from 10 to 15 min	1.2	0.20	2.6	0.31	1.1	0.12	<0.0001
from 15 to 30 min	1.9	0.38	5.6	0.62	1.4	0.23	<0.0001
from 30 to 60 min	2.8	0.58	5.5	0.65	2.0	0.23	<0.0001
from 0 to 60 min	5.4	0.87	13.3	1.53	5.5	0.59	<0.0001

^¶^ Time between the isoproterenol challenge and maximal response. ^Ø^ Area under the concentration curve and above baseline.

**Table 6:** Correlation coefficients * between basal plasma FFA and plasma FFA responses to the isoproterenol challenge in overfed, underfed or control *Merinos d’Arles* ewes (*n* = 36).

Item	Response at 5 min	Response at 10 min	Response at 15 min	Response at 20 min	Maximal Response	Time ^¶^	AUC ^Ø^ 0–60	AUC 0–5	AUC 5–10	AUC 10–15	AUC 15–30	AUC 30–60
Basal FFA	0.61	0.73	0.69	0.66	0.54	0.25	0.69	0.79	0.72	0.73	0.65	0.67
Response at 5 min		0.77	0.63	0.51	0.84	−0.08	0.67	0.96	0.92	0.72	0.52	0.44
Response at 10 min			0.91	0.85	0.83	0.30	0.91	0.83	0.96	0.98	0.84	0.72
Response at 15 min				0.97	0.77	0.43	0.99	0.70	0.84	0.98	0.98	0.82
Response at 20 min					0.72	0.47	0.97	0.61	0.75	0.93	1.00	0.89
Maximal response						−0.07	0.80	0.84	0.89	0.82	0.73	0.69
Time							0.40	0.01	0.15	0.37	0.47	0.44
AUC 0–60								0.74	0.86	0.97	0.97	0.83
AUC 0–5									0.94	0.79	0.61	0.59
AUC 5–10										0.92	0.75	0.64
AUC 10–15											0.93	0.79
AUC 15–30												0.89

* All significant at *p* < 0.0001, except for the variable time. ^¶^ Time between the isoproterenol challenge and maximal response. ^Ø^ Area under the concentration curve and above the baseline.
